# Exploring Cancer Experiences and Community Health Concerns Among Asian Americans in St. Louis: A Formative Study to Guide Photovoice Implementation

**DOI:** 10.1016/j.focus.2025.100470

**Published:** 2025-11-29

**Authors:** Bailey A. Martin-Giacalone, Sunny C. Lin, Jin E. Kim-Mozeleski, Xinyu Jade Gu, Brynn Mackenzie Lau, Bettina Drake, Erin Linnenbringer

**Affiliations:** 1 Division of Public Health Sciences, Department of Surgery, Washington University School of Medicine in St. Louis, St. Louis, Missouri; 2 Division of General Medical Sciences, Department of Medicine, Washington University School of Medicine in St. Louis, St. Louis, Missouri; 3 Prevention Research Center for Healthy Neighborhoods, Department of Population and Quantitative Health Sciences, Case Western Reserve University, Cleveland, Ohio

**Keywords:** Asian Americans, cancer health equity, photovoice, healthcare access

## Abstract

**Introduction:**

The objective of this study was to identify preliminary community concerns for cancer prevention and care among Asian Americans in the St. Louis metropolitan region.

**Methods:**

The authors used phenomenological theory to conduct an expressive writing study among Asian Americans who had a family or personal history of cancer or served as a cancer survivor’s caregiver. Participants handwrote essays about their experiences and perceptions of cancer. Three research team members coded the essays and generated themes using thematic analysis.

**Results:**

Nineteen individuals participated in the study. The authors identified 3 primary themes. First, cancer stigma was associated with the belief that lifestyle choices are the primary determinants of cancer. Second, preference for traditional medicine was influenced by different generational and cultural beliefs about how to treat cancer. Finally, the primary barriers to access to cancer care were financial cost and lack of language translation services.

**Conclusions:**

The authors identified several barriers to cancer prevention and care among Asian Americans, a population underrepresented in the St. Louis metropolitan region. These findings will guide participants’ engagement in a photovoice study aimed at improving community health.

## HIGHLIGHTS

• Expressive writing guided Asian Americans’ engagement in a photovoice study.
• Participants described that cancer stigma is rooted in individual blame for cancer.
• Preference for traditional medicine was influenced by generational beliefs.
• Financial cost and lack of translation services were key barriers to cancer care.

## INTRODUCTION

Cancer is the leading cause of death among Asian Americans.^1^ Despite the persistent model minority myth that portrays the Asian American population as inherently healthy and wealthy, Asian American communities have complex barriers to cancer prevention and care.^2^, ^3^, ^4^, ^5^ Similar to other groups who are socially and economically marginalized, structural barriers impacting Asian Americans include the lack of a universal healthcare system and limited language services, whereas specific cultural factors identified among Asian Americans include a preference for complementary or alternative medicine, family decision making around cancer care, and social stigma around cancer.^6^, ^7^, ^8^

Most Asian American health research has focused on populations in U.S. regions outside of the Midwest and states with larger proportions of Asian Americans.^9^ This has resulted in a knowledge gap of the health needs among Asian Americans living in Midwest communities where Asian Americans are underrepresented or significantly less than the national average of 7%.^10^ Asian Americans living in communities where Asian Americans are underrepresented may face additional or intensified barriers to healthcare access because of a greater lack of health-related resources, including community-based advocacy organizations, health fairs, and culturally competent social services.^11^^,^^12^ A focus on these communities could provide greater understanding of the range of experiences, exposures, and other determinants impacting cancer prevention and care among the overall Asian American population.

Given that there have been few studies on Asian Americans in Missouri, the authors sought to explore cancer-related community health needs of Asian Americans in the St. Louis Metropolitan Statistical Area, a community comprising 3.5% of the region’s population.^9^^,^^10^ This formative study also served to prepare participants for a follow-up community-based participatory research study method known as photovoice.^13^^,^^14^ Given that cancer is known to be a stigmatizing topic for Asian Americans to discuss in community settings, the authors used expressive writing as a qualitative method to give participants the opportunity to reflect on their cancer experiences before discussing them in group settings during photovoice.^15^^,^^16^

## METHODS

### Study Sample

This study was part of a larger photovoice study (The Asian Health Equity and Cancer Health Project) approved by the IRB at Washington University School of Medicine in St. Louis. The authors used purposive sampling to recruit individuals who self-identified as Asian, Asian American, or another term within the Asian diaspora and who either had a personal or family history of cancer or served as a caregiver of a cancer survivor. Eligibility criteria also included age ≥15 years and residence in the St. Louis Metropolitan Statistical Area. Youth and young adults were intentionally included in the study because they are known to be involved in shared decision making around health in Asian American families, cancer incidence is increasing among this population, and this age group is underrepresented in the health research despite having deep knowledge of health and being agents of change.^7^^,^^17^^,^^18^ Heterogenous sampling was broad among Asian ethnic identity, age group, gender, and generation of immigration to ensure a wide range of experiences among participants, while protecting participant confidentiality. The authors recruited participants by sharing study flyers with community organizations and advertising the study through the institution’s research recruitment services.^19^ Interested individuals completed a survey to confirm eligibility.

### Study Procedures

This study was conducted in English, and the authors used phenomenology as the study theoretical framework and study methodology. Expressive writing took place at Washington University School of Medicine in St. Louis in January 2024. Participants provided written, informed consent and completed a demographic survey. The authors adapted expressive writing methods by Lu et al.^20^ to fit the study question. As an introductory exercise to prepare participants for photovoice, the authors shortened the time from 20 minutes to 15 minutes, and the authors only implemented the first of 3 prompts by Lu and colleagues.^20^ The authors further modified the prompt to reflect general experiences rather than a cancer diagnosis: *write about your deepest thoughts and feelings regarding your perspectives of and experiences with cancer*. Participants individually responded to the prompt during 1 of the group meetings, and they were asked to respond in English, with pen and paper or until they had written at least 1 page.

### Data Analysis

The authors typed the expressive writing essays, which averaged 300 words (range=86–790). Prior to analysis, the authors—a coding team comprising 3 public health researchers trained in qualitative methods—engaged in a reflexivity exercise to limit influence of their biases, including reflection of their cultural and training backgrounds. Three of the authors conducted a thematic analysis of the essays and, aligned with phenomenological theory, analyzed the essays on the basis of 2 primary research questions: *What are their experiences?* and *In what contexts or situations did they experience it?*^21^ Individually, each of the authors hand coded the data using inductive coding. The authors reviewed codes together, created consensus of codes, and developed a codebook on the basis of agreement of code definition and examples. Two of the authors applied the final codes to the text, and the third person resolved discrepancies. The authors created categories from the codes and generated qualitative themes.

## RESULTS

Nineteen participants completed expressive writing (Table 1). Sixteen (84.2%) had a first- or second-degree family member with cancer, 2 (10.5%) had a personal history of cancer, and 1 (5.3%) was a caregiver; no participants reported inclusion in more than 1 of these categories. Three primary themes emerged from their expressive writing essays. First, cancer stigma was associated with the belief that lifestyle choices are the primary determinants of cancer. Second, preference for traditional medicine was influenced by different generational and cultural beliefs about how to treat cancer. Finally, the primary barriers to access to cancer care were financial cost and lack of language translation services (Table 2).

**Table 1:** Demographic Characteristics of Participants (N=19)

Characteristic	n (%)
Age group, years	
<18	2 (10.5)
18–30	9 (47.4)
30–49	5 (26.3)
50–65	3 (15.8)
Highest level of education	
Less than Grade 9	0 (0)
Some high school (Grades 9–12)	2 (10.5)
High-school graduate	0 (0)
Some college, no degree	2 (10.5)
Two-year associate degree	0 (0)
Four-year college or university degree	7 (36.9)
Master’s degree	2 (10.5)
Professional school degree	6 (31.6)
English proficiency	
Excellent	14 (73.7)
Good	4 (21.1)
Fair	1 (5.2)
Poor	(0)
Gender	
Man	6 (31.6)
Woman	13 (68.4)
Nonbinary or transgender	0 (0)
Place of birth	
U.S.	11 (57.9)
Outside U.S.	8 (42.1)
“How would you describe your racial, ethnic, or cultural identity?”^a^	
East Asia	4 (21.1)
South Asia	6 (31.5)
Southeast Asia	3 (15.8)
More than 1 above category	2 (10.5)
Another category not listed above	4 (21.1)

a This survey question was open ended, allowing participants to respond with terms that they used to describe themselves. The authors aggregated the terms into categories by large regional Asian ethnic groups to protect participant confidentiality.

**Table 2:** Study Themes and Representative Quotes

Theme	Representative quotes
Cancer stigma was associated with the belief that personal behaviors are the primary determinants of cancer	“The ‘taboo’ of acknowledging that I had cancer or people knowing that I had cancer was overwhelming… this disease is not my causing but was seen as [a] negatively impacting factor in [my] community” (Participant 10: a man, born outside the U.S., aged 50–65 years). “I was raised to believe if you ate the right things, did what the doctor said, and stayed active, you would live a healthy life. Only those who engaged in risky behavior, smoked, drank in excess, did illicit drugs, etc. would get [cancer]…” (Participant 15: a woman, born in the U.S., aged 30–49 years). “In my family, cancer is not a topic we normally talk about…it’s like an unsaid rule to not bring up bad things so that they don’t come true” (Participant 17: a woman, born in the U.S., aged 18–29 years).
Preference for traditional medicine was influenced by different generational and cultural beliefs about how to treat cancer	“Lack of understanding of healthcare… really affects the next generations. My grandma is very resistant to have modern medicine performed on her. This drastically increases the stress of those who have to take care of her (my mom and her siblings) and creates a… confused attitude towards healthcare” (Participant 14: a woman, born in the U.S., aged <18 years). “In a lot of Asian cultures, we tend to not trust doctors (especially western doctors) very much. This leads to my relatives trying to find other resources, which can often be misleading (Participant 13: a woman, born in the U.S., aged 18–29 years).
The primary barriers to access to cancer care were financial cost and lack of language translation services	“My first experience [with cancer] would be when my mother was diagnosed. Already advanced by diagnosis, [it] could have potentially been caught earlier with better healthcare availability/access. But my family was newly immigrated with little to no money at that time (Participant 16: a man, born in the U.S., age 18-29).” “We would all go [to my grandpa’s cancer care] appointments with him and have to help him translate what was being said to him by his doctors… I think something that could have helped more is better access and materials to healthcare information on cancer and also some way to help non-native English speakers… (Participant 18: a man, born in the U.S., age 18-29).”

In terms of the theme of social stigma around cancer, participants described “the ‘taboo’ of acknowledging that I had cancer…” and that “cancer is often [used to] curse others out or wish ill upon others.” Many expressed that cancer stigma exists because members of the community—and health insurance agencies—see cancer as an individual’s fault. One participant wrote, “[my] cancer diagnosis had me denied health insurance last year – what a travesty…it has been seen as if it’s my fault I got this (Participant 10: a man, born outside the U.S., age 50–65 years).” Several other participants emphasized that they grew up learning that the primary determinants of cancer are individual behaviors, such as smoking, drinking alcohol, and lack of exercise. However, some participants did describe people that they knew who actively avoided these behaviors yet still got cancer. For example, one participant, who was a healthcare provider, described treating patients with lung cancer, “many of whom had never smoked in their lives – which went contrary to what I had been led to believe as my health foundation” (Participant 15: a woman, born in the U.S., aged 30–49 years). Furthermore, social stigma around cancer led to the avoidance of discussing cancer in Asian families. One participant explained that after her mom was diagnosed with cancer, she “avoided discussing the topic with [my mom] directly because [of] …the culture of stifled emotions and expressions in my household and in other Asian families/communities…[that] deprived me of those important months in which I could’ve really been there for my mom” (Participant 1: a woman, born outside the U.S., aged 18–29 years).

Regarding the theme of preference for traditional medicine, participants of younger generations who were born in the U.S. often shared that they were conflicted or confused about the use of allopathic or Western medicine for treating cancer and other ailments. They wrote that these feelings often arose because older members of their families were hesitant to use Western medicine. However, one participant stated a different reason for not using Western medicine: “my friend’s father… had stomach cancer. His father relied heavily on ayurvedic (Indian herbal remedy) medicine to control the spread of cancer and pain management as allopathic medication made him feel worse…” (Participant 2: a woman, born outside the U.S., aged 30–49 years). Participants sometimes linked their view of Western medicine to political histories between the U.S. and their home country. For example, one participant wrote, “cancer is a relatively new development. My people…didn’t have cancer on the Islands when they were growing up. Cancer is a white man’s invention,’ referencing big Pharma” (Participant 19: a woman, born outside the U.S., aged 18–30 years).

In terms of the theme of barriers to cancer care, several participants discussed financial costs of care as a barrier to accessing services or paying for cancer treatment. Financial costs even led some families to leave the U.S. for care. For example, one participant shared that her mother, who had cancer “…went to [her home country] at some point to get care there and be with her family – although now I realize maybe money was a reason as well – given our expensive healthcare system” (Participant 6: a woman, born in the U.S., aged 30–49 years). Many participants also expressed that “language services have been very difficult to obtain.” In addition to the availability of in-language care, one participant suggested that their family did not trust translation services: “[my mom] would drive every week, three hours round trip to take my grandmother [who had cancer] to doctor’s appointments. My grandparents don’t speak English, so my mom was the translator, not wanting to rely on anyone else to do it” (Participant 9: a woman, born in the U.S., aged 18–29 years).

## DISCUSSION

Study findings highlight that cancer stigma, generational differences in preference for traditional medicine, and a lack of financial and language support services were primary concerns among participants in this study. Research has found that social stigma contributes to poor cancer screening rates among Asian Americans and worse quality of life outcomes among Chinese American breast cancer survivors.^16^^,^^22^, ^23^, ^24^ Thus, interventions aimed at helping Asian Americans manage social stigma around cancer are needed.^11^^,^^25^, ^26^, ^27^ Because the study findings highlight that social stigma around cancer is related to the belief that individual-level behaviors are the primary cause of cancer, future research might examine whether increasing cancer prevention knowledge on the environmental, social, and genetic risk factors for cancer reduce individual blame.

Participants also described how their family members had varied preferences for traditional and Western healthcare approaches, causing family stress and confusion. Complimentary medicine approaches are evidence-based practices that integrate traditional and Western medicine for a particular patient; research shows that these approaches may increase cancer screening and education and that some complementary therapies can reduce treatment-related toxicities and improve quality of life among patients with cancer.^28^, ^29^, ^30^ Future research could evaluate whether complementary medicine services are available among Asian American communities who are underrepresented in their geographic region and determine appropriate steps for implementing such services within specific community contexts.

This study also found that financial and language barriers to cancer education and care were major concerns among the study participants. Although National Cancer Institute–designated cancer centers offer financial and language assistance programs, it is possible that Asian Americans are not frequently connected with financial assistance owing to systemic assumptions that they do not face financial barriers.^5^^,^^31^^,^^32^ Studies examining access to language assistance have found that regions with smaller Asian American populations have a greater lack of such services as well as health-focused community organizations that help to educate and connect people to these programs.^12^ Therefore, future research could consider evaluating the reach of existing outreach efforts, such as cost-free cancer screening, and the availability and quality of language services in regions where Asian Americans are underrepresented.^11^^,^^32^

### Limitations

This study has limitations that should be considered. First, participants likely did not share all their experiences and perceptions of cancer because they only had 15 minutes to write. However, this study was intended to prepare participants for a photovoice study where these themes will be further explored. In addition, the authors did not disaggregate data by Asian ethnic subgroup because the photovoice study aims to generate policy solutions to community health needs. Therefore, the authors argue that this study serves as a case in which having an aggregate Asian American sample with diverse characteristics is appropriate, given that the origins of the term were to consolidate power for achieving a policy goal among individuals with shared lived experiences.^33^ In addition, the study sought to identify shared aspects of cultural identity and values across Asian American subgroups that shape the collective health experiences of Asian Americans.^34^^,^^35^ The authors were also unable to conduct subgroup analysis by participant relationship to cancer (survivor, family history, or caregiver) owing to small sample sizes within each group. Future research could consider examining subgroup-specific needs. Finally, participants in this study had high levels of education, reflecting the impact of the immigration preference system on the professional backgrounds of Asian Americans across generations and in St. Louis.^36^^,^^37^ As a qualitative investigation, this study is not intended to be generalizable and may not be representative of other Asian American populations.

## CONCLUSIONS

This study findings contribute to filling the gap in literature on the cancer health experiences and concerns of Asian Americans living in communities where Asian Americans are underrepresented. The authors plan to investigate these community concerns in a follow-up photovoice study that aims to develop community-driven solutions. Future directions also include conducting additional expressive writing studies to enhance credibility and trustworthiness of the themes and among different subgroups with unique cancer-related health needs. Given that many of the findings are systemic and structural factors, this work may provide additional insight for advancing health equity among other communities facing similar barriers to cancer prevention and care.
